# Sick leave among people in paid work after age 65: A Swedish population-based study covering 1995, 2000, 2005 and 2010

**DOI:** 10.1177/1403494817731487

**Published:** 2017-09-15

**Authors:** K. Farrants, S. Marklund, L. Kjeldgård, J. Head, K. Alexanderson

**Affiliations:** 1 Division of Insurance Medicine, Department of Clinical Neuroscience, Karolinska Institutet, Sweden; 2 Department of Epidemiology and Public Health, University College London, UK

**Keywords:** Sick leave, elderly people, labour force, extending working life

## Abstract

*Aims:* Extending working life into older age groups is discussed in many countries. However, there is no knowledge about how this affects rates of sick leave. The aim of this work was to investigate rates of sick leave among people in paid work after retirement age and if such rates have changed over time. *Methods:* Swedish nationwide register data on people aged >65 years and living in Sweden in 1995, 2000, 2005 and 2010 were analysed. All people with a sufficiently high work income to be eligible for public sick leave benefits were included. The proportions in paid work and compensated rates of sick leave for people aged 66–70 and ≥71 were analysed by sex, educational level, country of birth, living area, and employment type and sector. *Results:* The percentage of people in paid work at ages 66–70 years increased from <10% in 1995 to 24% in 2010 and among those aged ≥71 years from 2.7% in 1995 to 3.5% in 2010. The rates of sick leave among working people aged 66–70 years were 3.3% in 1995 and 2.4% in 2010 and for people aged ≥71 years the rates of sick leave were 2.2% in 1995 and 0.2% in 2010. Women had higher rates of sick leave than men in 2005 and 2010, but lower in 1995 and 2000. In 2010, the rates of sick leave were similar between employees and the self-employed, and higher among employees in the public sector than among employees in the private sector. ***Conclusions:* Rates of sick leave among workers aged >65 years were lower in 2010 than in 1995, despite much higher rates of labour market participation in 2010**.

## Background

Improving the possibilities for older people to remain longer in paid work and raising the retirement age have been discussed in many countries [1,2]. Although such intentions are mainly economically motivated, an increasing number of people working at older ages may affect public health and national levels of sick leave [3,4] because an older age is generally linked to higher morbidity [5] and sick leave [6,7]. An important question is thus whether rates of sick leave will increase when more people of older ages remain in paid work. Little research has focused on sick leave among older employees and, to our knowledge, no study has been published regarding sick leave among people who continue to work after the standard retirement age.

Several studies have found associations between age and risk of sick leave. A literature review from 2009 concluded that younger people have a higher frequency of sick leave, but older people have longer periods of sick leave [6]. Brenner and Ahern [8] showed that the oldest age group reported significantly more absence from work on health grounds. Older age has also been found to be related to an increase in the duration of sick leave due to mental disorders [9]. However, another study showed that sick leave with common mental disorders was more frequent among young people, but that there was no significant age difference in the risk of recurrent periods of sick leave [10]. A study based on labour force surveys from nine countries concluded that an older age was generally associated with higher rates of sick leave, but this was partly related to longer employment tenure in older age groups [11]. A Brazilian study showed higher risks of short- and medium-term sick leave among younger cohorts [12]. Because there is great variation among measures of sick leave in published papers, these differences might be due to the choice of measure [13]. Also people in the age groups closer to retirement may have lower rates of sick leave than middle-aged people due to health-related selection out of the labour market and into early retirement or disability pension [14,15].

A systematic literature review of factors related to sick leave concluded that few studies have analysed the effect of age on sick leave in detail [7]. Some researchers have studied whether age is systematically associated with factors related to sick leave. Taimela et al. [15] showed that young workers had a higher annual prevalence of sick leave despite better self-reported health than older workers. Arola et al. [16] found no significant effect of age in the association between job control and sick leave. Conversely, downsizing effects on sick leave were stronger among older workers [7]. A Dutch study [17] on the associations of work and family life on sick leave found that employees aged 45–55 years who reported a conflict between work and private life had a higher risk of prolonged sick leave, whereas this factor affected employees younger than 36 years to a lesser degree and those older than 55 years not at all.

Increasing numbers of people stay in work up to and beyond standard pension age [18]. Different types of selection make older workers distinct from those who exit earlier from the labour market [4,19]. Some people return to paid work after a period of retirement [20]. People in white collar occupations and occupations that require a university education are more likely to work after retirement age [21], as are self-employed people [22]. People with health problems and restricted work capacity more often choose to retire early, whereas those without such problems continue to work [21]. Stenholm et al. [23] found that physical functioning declined faster among retired people after 65 years of age than among full-time employed people of the same age. The selection bias among older workers moving into disability pension makes the age differences in sick leave smaller than they would otherwise be [7].

We currently lack even a basic knowledge of rates of sick leave among those who work after the age of 65 years and whether the rates of sick leave among this group have changed over time. This is the first study of sick leave in this group and is thus an exploratory study. The aim of this study was to describe numbers of people aged 66–70 and ≥71 years still engaged in paid work and their sick leave in four different time periods (1995, 2000, 2005 and 2010) and the sociodemographic and employment characteristics of people taking sick leave.

## Methods

### Design

We used four cross-sectional studies to investigate the amount of sick leave during one whole year for each of four large population-based groups covering all people registered as living in Sweden during the years 1995, 2000, 2005 and 2010.

### Material and study population

The study was based on data from Statistics Sweden. All people registered as living in Sweden who were at least 65 years of age on 31 December of the years 1994, 1999, 2004 and 2009 were selected for one of the four respective groups. Information on all their sickness absence compensated by the National Social Insurance Agency was obtained for the years 1995, 2000, 2005 and 2010.

The statutory age for an old-age pension in Sweden was traditionally 65 years, but it can be taken earlier. Waiting to take out a pension will increase the value of the pension. Since 2001, people have had the statutory right to keep their permanent employment until the age of 67 years, after which it is up to the employer whether to continue the employment. To be able to compare the rates of sick leave among those older than 65 years with those younger than 65 years, the corresponding figures for people aged 60–64 years for the four respective years were also obtained.

All people in Sweden in paid work who have reduced work capacity due to disease or injury can receive sick leave benefits from the public sickness insurance system; however, there are some restrictions from the age of 65 years. People aged between 65 and 69 years can obtain sick leave benefits for up to a total of 180 days during those years, after which the social insurance agency may restrict further claims if the reduced work capacity is assessed as permanent. From the age of 70 years, people cannot claim sick leave benefits for >180 days.

People with a permanently reduced work capacity are expected to live on their retirement pensions rather than on sick leave benefits. In the period of this study, the maximum level of sickness benefit was about 80% of lost income, up to a maximum of 7.5 times the national price basic amount (a set amount that changes with inflation each year and is used as the basis for indexation). In the year 1995, the price basic amount was about €3896 and in 2010 it was about €4626 (using the 2010 conversation rate between Swedish krona and euros for both figures).

### Variables

Information on age, sex, employment, level of work income, sick leave, educational level, birth country and living area was obtained from the longitudinal integration database for health Insurance and labour market studies (LISA). The variables ‘in paid work after 65’ and ‘compensated sick leave’ were used.

Work income was used as the main selection variable to define the target population of people who could potentially be sickness absent. A minimum annual work income of 24% of the price basic amount was required to be eligible for public sick leave benefit. Work income is derived from labour market participation and excludes income from capital, pensions or other sources. The minimum income requirement in the selection was adjusted to 75% of the minimum annual work income for sick leave benefit because sick leave benefit in most instances covers 75% of the lost income. Without this adjustment, people with a low income who had longer periods of sick leave might have been excluded. In this study, ‘working after 65’ was defined as people who met this requirement and ‘not working after 65’ included those who did not meet this requirement.

In Sweden, during the years studied, sick leave was compensated by the employer during the first two weeks of a period of sick leave, excluding the first day, which was a qualifying day without compensation. A period of sick leave lasting for 15 days or longer was compensated through the National Social Insurance Agency. Self-employed people were compensated by the National Social Insurance Agency for the entire period of sick leave, excluding qualifying days. Self-employed people could choose a number of qualifying days from 3 to 30 for most of the study period, with an upper limit of 90 days in 2010, and pay for the insurance accordingly, but after the age of 55 years they could not reduce their number of qualifying days. This study only includes information on sick leave compensated by the National Social Insurance Agency.

We calculated the following measures of sick leave during one year among those in paid work: the number and rates of people with at least one compensated period of sick leave; the number of compensated periods of sick leave per person; and the rates of people with at least one compensated period of sick leave of duration 1–14, 15–28, 29–60, 61–180 and 181–365 days, respectively. Table I gives information on educational level, type of employment, employment sector, type of living area and country of birth.

**Table I.** Number of people still in paid work aged 66–70 and ≥71 years, divided by sex, country of birth, type of living area, education, type of employment and employment sector in 1995, 2000, 2005 and 2010.

Age group (years):	66–70				≥71			
Year:	1995	2000	2005	2010	1995	2000	2005	2010
*All*	38,202	40,836	66,222	121,091	23,856	26,345	35,929	38,680
*Sex*								
Women	13,045	14,797	26,339	49,584	6922	7935	11,528	12,472
Men	25,157	26,039	39,883	71,507	16,943	18,410	24,401	26,208
Missing	0	0	0	0	0	0	0	0
*Country of birth*								
Sweden	35,378	37,495	60,289	111,520	22,582	24,619	33,410	35,891
Other Nordic country	1268	1595	2835	4114	561	725	1092	1230
Other country	1556	1745	3098	5456	721	1001	1425	1559
Missing	0	1	0	1	1	0	2	0
*Living area*								
Large city (Stockholm, Gothenburg or Malmö)	13,468	14,973	23,663	43,602	8909	9644	12,153	11,958
Medium-sized city (>90,000 inhabitants)	12,490	13,410	22,309	41,577	7601	8749	11,910	12,860
Small city/villages (<90,000 inhabitants)	12,244	12,453	20,250	35,912	7355	7952	11,866	13,862
Missing	0	0	0	0	0	0	0	0
*Education level*								
Primary (≤9 years)	15,962	15,177	20,619	30,352	6602	5902	7134	13,864
Secondary (10–12 years)	12,259	13,674	23,996	47,222	4904	4702	6529	13,472
Tertiary (≥13 years)	9771	11,849	21,401	43,171	3253	401	5964	11,192
Missing	210	136	206	346	165	11,729	90	152
*Type of employment*								
Employed	29,124	30,971	50,101	91,541	17,730	19,531	24,185	18,461
Self-employed	9078	9865	16,121	29,550	6135	6814	11,744	20,219
Missing	0	0	0	0	0	0	0	0
*Employment sector*								
State authority	4436	2163	2339	4538	2772	1090	441	343
Regional authority	1049	1082	2211	4552	291	257	284	357
Municipality	4993	5343	6707	12,465	2159	2425	1547	1104
Private sector	27,724	32,248	30,641	60,109	18,643	22,573	13,443	18,224
Missing	0	0	24,324	39,427	0	0	20,214	18,652

## Results

### Work and sick leave among people aged 66–70 and ≥71 years in 1995, 2000, 2005 and 2010

The number (Table I) and rate (Table II) of people aged 66-70 years in paid work was progressively higher in Sweden in each group studied from 1995 to 2010. Although the majority of those aged 66–70 did not have paid work, the rate of those who had paid work increased from <10% in 1995 to 24% in 2010. The proportion of people aged ≥71 years in paid work was low, but also increased from 2.7% in 1995 and 2000 to 3.5% in 2010 (Table II).

**Table II.** Rates (%) of people aged 66–70 and ≥71 years by work income in euros (adjusted for inflation using the 2010 price basic amount).

Adjusted income groups (in euros)	1995	2000	2005	2010
*All aged 66–70 years*	*n*=394,276	*n*=373,326	*n*=401,573	*n*=504,653
No work income	82.9	82.4	76.5	69.1
Low work income (€10–750)	7.3	6.7	7.0	6.9
Not working = no or low work income	90.2	89.1	83.5	76.0
751–999	0.8	0.9	1.0	1.1
1000–3999	4.5	4.5	5.8	6.7
4000–7999	2.0	2.3	3.2	4.4
8000–15,999	1.5	1.9	2.9	5.1
16,000–29,999	0.7	0.9	2.4	4.0
≥30,000	0.3	0.4	1.2	2.6
Working = work income	9.8	10.9	16.5	24.0
*All aged ≥71 years*	*n*=884,594	*n*=980,898	*n*=1,025,926	*n*=1,083,661
No work income	93.7	94.1	92.9	94.6
Low work income (€10–750)	3.6	3.2	3.6	1.9
Not working = no or low work income	97.3	97.3	96.5	96.5
751–999	0.3	0.3	0.4	0.3
1000–3999	1.4	1.4	1.7	1.4
4000–7999	0.5	0.5	0.7	0.7
8000–15,999	0.3	0.3	0.4	0.7
16,000–29,999	0.1	0.1	0.2	0.3
≥30,000	0.0	0.1	0.1	0.1
Working = work income	2.7	2.7	3.5	3.5

Table III shows that the number of working people aged 66–70 years has more than trebled from <40,000 in 1995 to >120,000 in 2010. The rate of increase was stronger in the later years. The number of working people aged ≥71 years was lower, but it also increased between 1995 (about 24,000) and 2010 (about 40,000). By contrast, among those aged 66–70 years in work, the proportion with any sick leave benefits was higher in 1995 (3.3%) than in 2010 (2.5%). Among those aged ≥71 years and in work, the proportion with sick leave decreased even more from 2.2% in 1995 to 0.2% in 2010. The number of people in paid work was much higher among those aged 60–64 years than among those aged 66–70 or ≥71 years in all four years studied (220,485 in 1995, 253,104 in 2000, 383,194 in 2005 and 429,367 in 2010). The rates of sick leave in the age group 60–64 years were substantially higher in all years than for those aged >65 years (14.9% in 1995, 16% in 2000, 13.1% in 2005 and 10.9% in 2010). In this age group, the rates of sick leave increased between 1995 and 2000, unlike the rates of sick leave of the older groups, but after 2000 they also decreased in the younger age group.

**Table III.** Number (%) of people aged 60–64, 66–70 and ≥71 years with and without compensated sick leave of >14 days during 1995, 2000, 2005 and 2010.

Year	Total working			No sick leave			Sick leave			No sick leave (%)			With sick leave (%)		
	60–64 years	66–70 years	≥71 years	60–64 years	66–70 years	≥71 years	60–64 years	66–70 years	≥71 years	60–64 years	66–70 years	≥71 years	60–64 years	66–70 years	≥71 years
1995	220,485	38,202	23,865	187,717	36,921	23,350	32,768	1281	515	85.1	96.7	97.8	14.9	3.3	2.2
2000	253,104	40,836	26,345	212,525	39,804	26,052	40,579	1032	293	84.0	97.5	98.9	16.0	2.5	1.1
2005	383,194	66,222	35,929	333,126	64,562	35,653	50,068	1660	276	86.9	97.5	99.2	13.1	2.5	0.8
2010	429,367	121,091	38,680	382,661	118,189	38,454	46,706	2902	226	89.1	97.6	99.4	10.9	2.4	0.2

### Number of periods of sick leave

**Figure 1.**
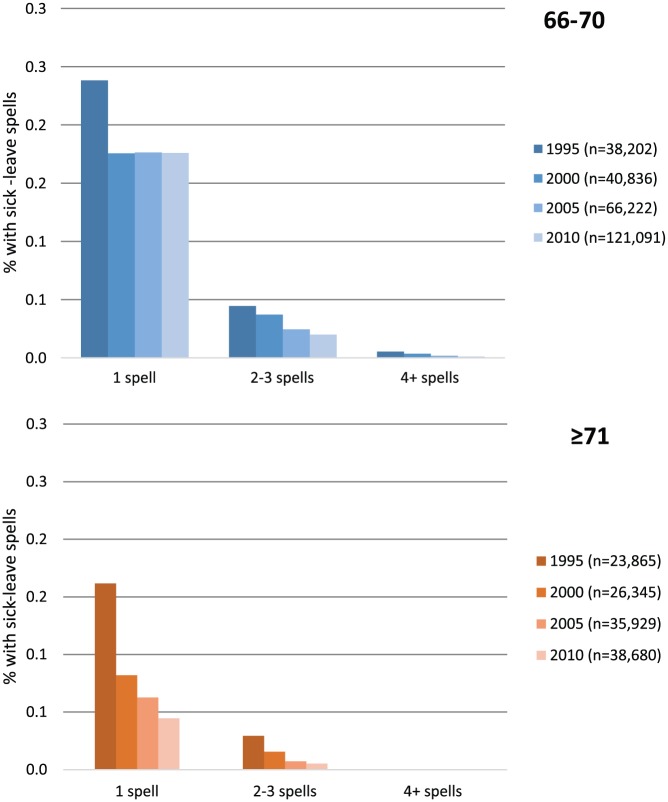
Rates (%) of people aged 66–70 and ≥71 year who had had one or more period of sick leave >14 days compensated by the Social Insurance Agency in 1995, 2000, 2005 and 2010.

Figure 1 shows that most of those with sick leave had only one period of sick leave during the study year. Hardly anyone had four or more periods of sick leave. In both age groups there were progressively fewer periods of sick leaves in each group from 1995 to 2010 and the largest change took place between 1995 and 2000.

### Number of periods of sick leave of different durations

**Figure 2.**
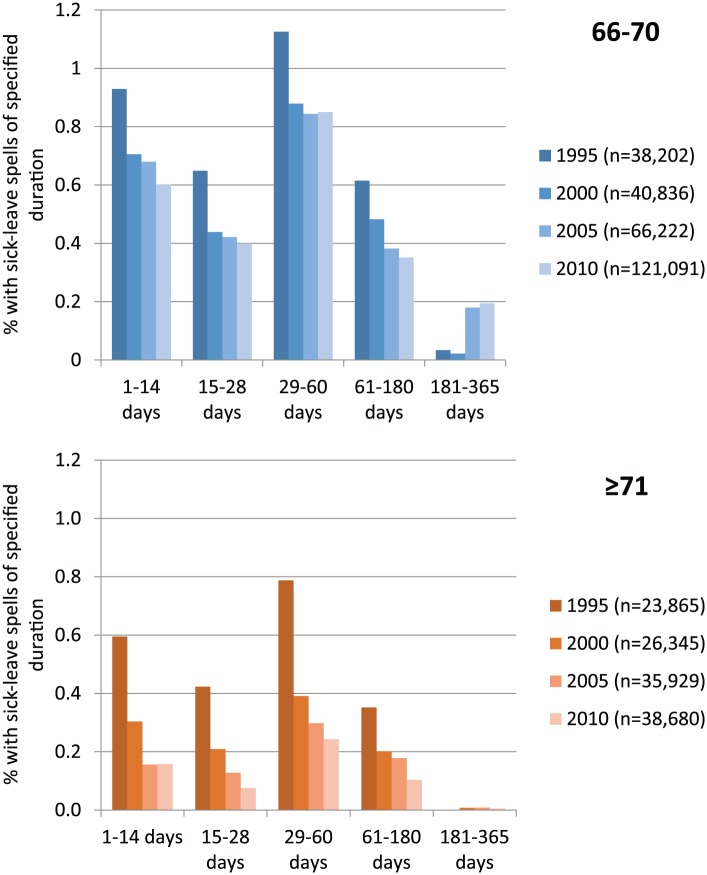
Rates (%) of people aged 66–70 and ≥71 years with periods of sick leave >14 days compensated by the Social Insurance Agency of a certain duration in 1995, 2000, 2005 and 2010.

The decrease in sick leave can also be seen by the duration of sick leave (Figure 2). Among those aged 66–70 years there was a decrease in all durations from 1995 to 2010, except those few periods of sick leave lasting ≥181 days. In all four years the most common duration of a period of sick leave was 29–60 days. In the older age group the number of periods of sick leave of all different durations was lower and there was no period ≥181 days.

### Sociodemographic and employment characteristics of people with sick leave

Table IV shows that there was a higher rate of men aged 66–70 years with sick leave in 1995 and 2000, whereas in 2005 and 2010 the rate of women with sick leave was higher. The same sex shift took place among those aged ≥71 years. The rates of sick leave among people born outside the Nordic countries were generally higher than for those born in Sweden or another Nordic country, especially among people ≥71 years of age. Rates of sick leave were similar in different types of living areas, but metropolitan areas had higher rates of sick leave in both age groups and in all four years. Rates of sick leave in the three educational categories, in both age groups, became more similar over time. In 2010, those with the highest level of education had higher rates of sick leave in both age groups, which was not the case in earlier years (Table IV).

**Table IV.** Rates (%) of people with sick leave benefit among those working after the age of 65 years by age group, sociodemographic group, type of employment and sector of employment.

Age group (years):	66–70				≥71			
Year:	1995	2000	2005	2010	1995	2000	2005	2010
*N*	38,202	40,836	66,222	121,091	23,865	26,345	35,929	36,680
All	3.3	2.5	2.5	2.4	2.2	1.1	0.8	0.6
*Sex*								
Women	3.2	2.4	2.6	2.6	1.9	0.9	0.9	0.5
Men	3.4	2.6	2.4	2.3	2.2	1.2	0.7	0.6
*Country of birth*								
Sweden	3.3	2.5	2.4	2.3	2.0	1.0	0.6	0.5
Nordic country	3.0	2.5	2.7	2.6	3.2	0.5	1.5	0.6
Other country	4.6	2.9	3.6	3.3	4.1	2.5	1.3	1.7
*Living area*								
Large city	3.6	2.7	3.0	3.0	2.5	1.4	1.0	0.8
Medium-sized city	3.4	2.2	2.4	2.3	2.0	1.0	0.7	0.5
Small city/village	3.2	2.4	2.0	1.8	1.8	0.6	0.6	0.4
*Education level*								
Primary	3.5	2.6	2.2	2.1	2.3	1.1	0.8	0.4
Secondary	3.3	2.6	2.6	2.3	2.3	1.3	1.2	0.6
Tertiary	2.8	2.0	2.2	2.3	2.4	1.1	0.7	0.7
*Type of employment*								
Employed	2.0	1.4	2.2	2.2	1.4	0.8	0.5	0.5
Self-employed	7.5	5.7	3.0	2.3	4.1	2.0	1.1	0.5
*Employment sector*								
State authority	1.6	1.0	3.5	3.6	1.6	0.5	0.7	0.7
Regional authority	1.6	1.7	4.2	4.7	1.8	0.6	0.5	2.8
Municipality	1.8	1.0	4.1	4.3	1.6	2.3	1.1	1.1
Private sector	4.0	2.8	4.1	3.5	3.9	1.2	1.8	0.7

Table IV also shows the type and sector of employment among people with sick leave. There were much lower rates of sick leave among employed people than among the self-employed in both age groups in 1995. Rates of sick leave for self-employed people were also higher in 2000, but in 2005 and 2010 the dissimilarities attenuated. Private sector employees had higher rates of sick leave than those in other sectors in 1995, 2000 and 2005 in the age group 66–70 years, but they had lower rates than public sector employees in 2010 (Table IV). In those aged ≥71 years, employees from municipal or regional authorities had higher rates of sick leave than employees from other sectors in 2000 and in 2010.

## Discussion

In this first explorative study of sick leave in people older than retirement age, the first question concerned changes in the rate of the population in Sweden who worked after the usual pension age. Between 1995 and 2010 there was a progressively higher rate of people aged >65 years who had paid work, at least to the extent that they qualified for sick leave benefits, yet the rates of sick leave decreased over the period 1995–2010. The rates of multiple periods of sick leave and long periods of sick leave also decreased over time.

There are several reasons behind the identified increase of people in paid work after 65 years of age. Life expectancy has increased remarkably in most of the world in the last few decades and higher proportions of the population survive for many years after the standard retirement age [24]. This has led to a number of different challenges for societies [18,24]. Research suggests that people are living longer without severe disability [25,26], but also that the public cost of old-age pensions have increased substantially in most developed countries [27]. Thus in most welfare states with developed public systems for old-age pensions, discussions on the fiscal, economic and social needs for increasing the pension age are ongoing [1]. In Sweden, this took the form of a public pension reform in 2001. This reform introduced flexible retirement instead of a standard retirement age, as well as a change of how pension benefits were calculated, to strengthen the economic incentives to postpone retirement [28]. It is also possible to claim full pension benefits while still working full-time or being on sick leave.

In 2005 and 2010, Swedish baby-boomers – people born between 1940 and 1945 – had reached retirement age. This cohort is characterised by a high labour market participation, particularly among women, a high educational level, a high rate of white collar employees and a high rate of self-employment [18]. Both higher educational levels and the increase in self-employment have contributed to higher employment rates after the age of 65 years. The general trend of improved health in the older population and indicators of improved physical work environments may also have contributed to this development [24,25,29]. It has also been shown that there are large socio-economic differences in health and in the retirement decision [29]. It has also been projected that the prevalence of morbidity and mortality in the older population in Sweden will be reduced due to increasing numbers of people with higher education [30].

The study also showed that the rate of sick leave among people in paid work after the age of 65 years was low compared with those aged 60–64 years and decreased during the period 1995–2010 in both age groups studied. As this is the first study of sick leave in these older age categories, we do not have any comparable information about sick leave among people above retirement age from other countries or at other points in time. Neither are explanations for the decrease in sick leave in Sweden among people who have reached retirement age known. The decrease reflects a general decline in rates of sick leave during this time, as seen in our data for people aged 60–64 years, as well as in other studies of general sick leave in the entire population [31]. The reasons for the decrease in rates of sick leave are not known among the general population either; however, the numbers of people in paid work did not increase as dramatically as in those older than 65 years of age. The relatively low level of sick leave among people in paid work after the age of 65 years is most likely related to health selection in the choice to retire, where people with better health may more often choose to continue to work after retirement age. It may also be related to the possibility of less physically or mentally demanding occupations and working conditions, or more flexible or lower working hours, among those who continue to work compared with those who retire. There may also be an effect of tighter eligibility in the sickness insurance and earlier interventions implemented in 2008, including specifying time limits for when interventions should occur, leading to fewer or shorter periods of sick leave [32]. Nevertheless, when more people remain in paid work the health selection effect could be hypothesised to be lower and rates of sick leave to increase. This did not happen, indicating that more people might leave work when they have long-lasting health problems in the later cohorts than in the earlier cohorts. This deserves further study.

Concerning the sociodemographic characteristics among those with sick leave, this study showed a higher rate of sick leave among women in paid work after retirement age in 2005 and 2010, but a higher rate among men in 1995 and 2000. This shift may partially be related to the labour market and demographic characteristics of the age groups. The rates were similar between educational level and type of living area and became more so over the course of this study. In 1995, there were lower rates among employed than among self-employed people as well as among those who were employed in the public sector than among those employed in the private sector; however, the groups became more similar over time. This might possibly be because self-employed people choose longer waiting periods before they receive compensation from the National Insurance Agency, meaning that they do not show in our data, but might still be sick. Differences in the possibility of obtaining sick leave benefits between the two age groups in the different years may, to some degree, account for the changes in rates of sick leave by employment type sector. However, the introduction of stronger economic incentives and a statutory right for employees to work until the age of 67 years in the new pension system of 2001 may also have contributed to these results.

### Strengths and limitations

There are two main strengths of this study: it is the first of its kind and it is based on four population-based groups including almost all people older than 65 years living in Sweden during four different years. Other strengths are the large study groups, allowing for subgroup analyses, high-quality register data (not relying on self-reporting) and no loss to follow up. As the study was based on public register information, there are limited problems of selection bias or drop-out. It can be seen as both a strength and a limitation that only periods of sick leave with benefits from the Social Insurance Agency, mainly those >14 days, were included, because (for example) short-term infections were excluded. This means that we capture the prevalence of long-term, often chronic, sick leave, but not the shorter periods. We also miss periods of sick leave by self-employed people who have long waiting times before they receive benefits, although some self-employed people with periods of sick leave <14 days are included if their waiting time is shorter. This might potentially introduce bias in the results for sick leave among the self-employed.

As increasing rates of people above the standard retirement age continue in paid work and therefore might need sick leave, there is a strong need for more research in this area. Such studies could also include information on other factors of importance for remaining in paid work and for sick leave, such as health, working conditions and environment, and family situation.

## Conclusions

The numbers and rates of people in paid work after 65 years of age increased greatly in Sweden between 1995 and 2010. Despite this increase, the rates of sick leave of those in work after the age of 65 years decreased.
